# Development of immunochromatographic device as a point-of-care tool for serodiagnosis of human strongyloidiasis cases

**DOI:** 10.1007/s10096-019-03745-2

**Published:** 2019-11-22

**Authors:** Lakkhana Sadaow, Oranuch Sanpool, Rutchanee Rodpai, Patcharaporn Boonroumkaew, Wanchai Maleewong, Pewpan M. Intapan

**Affiliations:** 1 Department of Parasitology and Excellence in Medical Innovation, and Technology Research Group, Faculty of Medicine, Khon Kaen University, Khon Kaen, Thailand; 2 Research and Diagnostic Center for Emerging Infectious Diseases, Mekong Health Science Research Institute, Khon Kaen University, Khon Kaen, Thailand

**Keywords:** Diagnosis, Immunochromatographic test, Screening test, Rapid test, Strongyloidiasis, *Strongyloides stercoralis*

## Abstract

Human strongyloidiasis is an important gastrointestinal disease with an estimated 30 to 100 million people infected. Prevalence is generally underestimated since many infections are asymptomatic, and traditional diagnostic tests based on parasitological examination of stool samples are not adequately sensitive. Serological tests are useful and supportive but are still only available in a reference research setting. We made an immunochromatographic test (ICT) kit for rapid serodiagnosis of human strongyloidiasis. The antigen used in the ICT kit was extracted from larvae of *Strongyloides stercoralis*. Diagnostic efficacy of the kit was evaluated using human serum samples from strongyloidiasis patients, healthy persons, and those with other parasitoses. When using a cutoff level of 0.5 or above, the diagnostic sensitivity, specificity, and positive and negative predictive values at the prevalence of infection of 34.4%, were 93.3%, 83.7%, 76.7%, and 95.6%, respectively. This ICT kit is easy to use at the point-of-care and a result can be obtained in 15 min. Sophisticated instruments and highly trained staff are not required. It can be used in several diagnostic and public-health settings, e.g., prevalence surveys in endemic areas, confirmation and monitoring of cure post-treatment, diagnosis and screening of infected but asymptomatic individuals, and populations “at risk” for hyperinfection syndrome or disseminated strongyloidiasis if they are given immunosuppressive treatment for other conditions.

## Introduction

Human strongyloidiasis, a soil-transmitted helminthiasis, is a harmful intestinal parasitic disease that is distributed globally and infects an estimated 30 to 100 million people [1–4]. *Strongyloides stercoralis* is the main causative agent, while *Strongyloides fulleborni* has been reported from people in some parts of Africa, Papua New Guinea, and Thailand [1, 5]. The true number of infections is likely underestimated due to the low sensitivity of diagnostic tools delays in diagnosis. Three factors lead to delayed diagnosis: subclinical cases which remain unsuspected and undetected, irregular larval output in the feces making diagnosis difficult, and the lack of a gold-standard diagnostic test [4, 6].

The disease is generally diagnosed by detecting parasites in stool samples using methods such as direct fecal smear examination, Baermann concentration, formalin-ethyl acetate concentration, Harada-Mori filter paper culture, and agar plate cultures [4, 7]. However, these methods are complicated, require skilled microscopists, and are time-consuming. Furthermore, intermittent releases of worms in feces dictate the need for multiple stool examinations [8]. Molecular techniques such as polymerase chain reaction (PCR) and real-time PCR, for the detection of *Strongyloides* DNA in stool or urine samples, have been reported [9–14]. Serological assays (to detect antibody or antigen) can be used to provide support for a diagnosis [15–17]. For example, immunoblotting has been used to detect specific antibody against various antigenic polypeptide bands [18–24]. Recently, Yunus et al. [25] reported the developed lateral flow dipstick test using recombinant protein antigens for detection of human IgG4 antibody has been reported and the results showed high diagnostic sensitivity and specificity. However, these methods are also time consuming and require specialized equipment not generally available at the point-of-care (POC), and some methods require sophisticated and expensive instruments only found in advanced laboratories.

Here, we describe a new strip device, the rapid diagnostic immunochromatographic test (ICT) using somatic *S. stercoralis* larval soluble extract antigen for detection IgG antibodies in human sera. This POC test is simple and can be performed at the bedside for serodiagnosis of human strongyloidiasis.

## Materials and methods

### Parasites and antigens

*Strongyloides stercoralis* third-stage larvae were obtained from fecal samples from infected patients using the filter paper culture technique [26]. Larvae were washed several times in distilled water and then stored at − 20 °C until use. The frozen sample, packed with *S. stercoralis* L3 (500 μL), was homogenized with a tissue grinder in a small volume of distilled water containing 0.1 mM phenylmethylsulfonyl fluoride and 0.1 μM *N*-(*N*-[L-3 trans-carboxyoxiran-2-carbonyl]-L-leucyl)–agmatine. The extraction was then sonicated with an ultrasonic disintegrator and centrifuged at 10,000×*g* for 30 min at 4 °C. The supernatant was assayed for protein concentration using the Quick Start Bradford Protein Assay (Bio-Rad Laboratories Inc., CA). The somatic larval soluble extract was kept as the source of antigen and stored at − 70 °C until used.

### Human sera

Human serum samples were used for evaluation of the diagnostic value of the test. These sera were supplied by the frozen sample bank (− 70 °C) at the Faculty of Medicine, Khon Kaen University. The samples were divided into three groups: (i) the negative control group (*n* = 30) comprised samples from healthy adult volunteers who were free (based on stool examination) [27] from any intestinal parasitic infection at the time of blood collection; (ii) the strongyloidiasis group (*n* = 60), which comprised samples from parasitologically confirmed strongyloidiasis patients (based on the agar-plate culture method) [28], and (iii) serum samples (*n* = 74) from patients with parasitic infections other than strongyloidiasis (Table 1). The infections were verified by parasitological methods, except in cases of cysticercosis which were diagnosed using a computerized tomography scan and an immunological method [29] (Table 1). Pooled serum samples from the healthy individuals and from strongyloidiasis patients were also used as negative and positive controls, respectively. Precision of the method was investigated by performing the test on the same samples on different days: no day-to-day variation was seen. The study protocol was approved by the Khon Kaen University Ethics Committee for Human Research (HE611507).

**Table 1:** Human sera studied and results of the immunochromatographic test kit using somatic antigen extracted from *Strongyloides stercoralis* larvae

No.	Type of sera	Number of positive/total number
1	Proven strongyloidiasis	56/60
2	Healthy controls	0/30
3	Opisthorchiasis	0/5
4	Hookworm infections	1/5
5	Ascariasis	0/5
6	Taeniasis	2/5
7	Trichuriasis	2/5
8	Trichinellosis	0/5
9	Giardiasis	1/5
10	Amoebiasis	1/5
11	Blastocystosis	1/5
12	Cysticercosis	0/5
13	Angiostrongyliasis	2/5
14	Gnathostomiasis	1/5
15	Sparganosis	2/4
16	Capillariasis	2/5
17	Fascioliasis	2/5
	Sensitivity (%)	93.3
	Specificity (%)	83.7
	Positive predictive values (%)	76.7
	Negative predictive values (%)	95.6

### Immunochromatographic test kit

**Fig. 1:**
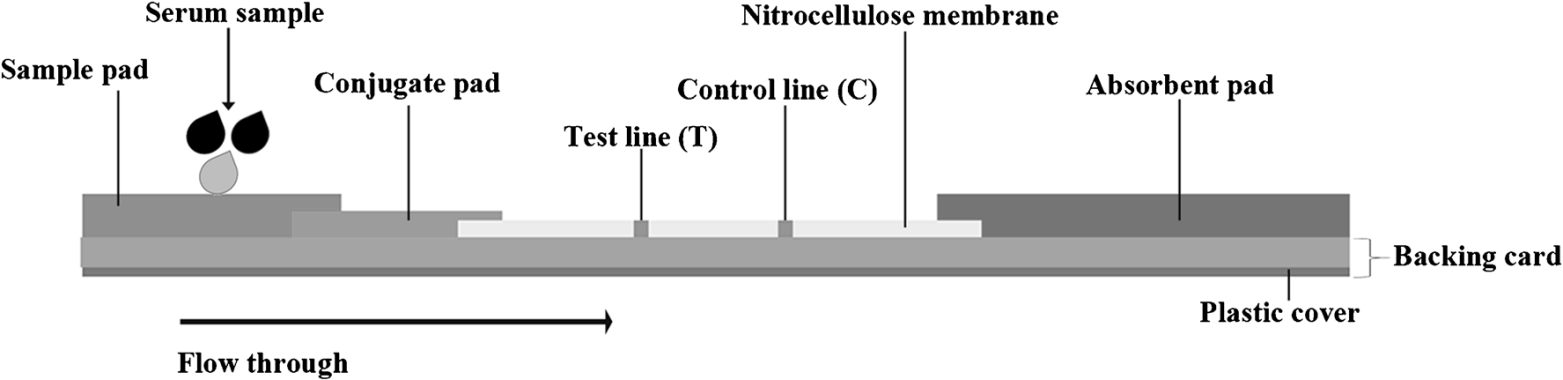
Schematic diagram of the immunochromatographic strip. The sample pad, conjugate pad, immobilized nitrocellulose membrane, and absorbent pad were stuck together on a plastic backing card. At the test line (T), the *Stercoralis stercoralis* antigen (1 mg/mL**)** and control line (C), the anti mouse IgG were fixed on the nitrocellulose membrane

**Fig. 2:**
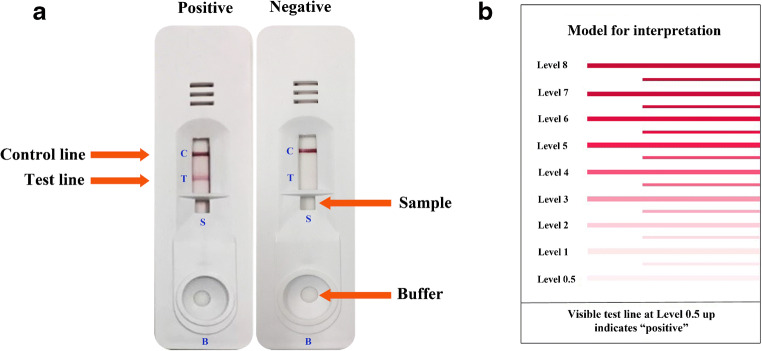
The immunochromatographic device for diagnosis of strongyloidiasis. **a** Example of a positive result on the ICT strip (left) and negative result (right). **b** The interpretation card for determining the cutoff level

The rapid ICT kit (named “strongyloidiasis ICT kit”) was optimized using somatic antigen extracted from larvae of *S. stercoralis*. Schematic diagram of ICT kit production was shown (Fig. 1). The test strip itself was based from a nitrocellulose membrane (Sartorius Stedim Biotech SA, Goettingen, Germany). Goat IgG anti-mouse IgG (Lampire Biological Laboratories) (0.1 μL/mm) was sprayed using XYZ3210 Dispense Platform (BioDot, Irvine, CA) onto the membrane to form the control line (“C” in Fig. 2a). Similarly, *S. stercoralis* antigen (1 mg/mL) was sprayed as the test line at a flow rate of 0.1 μL/mm (“T” in Fig. 2a), and colloidal gold-conjugated mouse monoclonal anti-human IgG (Kestrel BioSciences Co., Pathumthani, Thailand) was sprayed onto a piece of glass microfiber filter GF33 (Whatman Schleicher & Schuell, Dassel, Germany) to form the conjugate pad (Fig. . 1). Also required are sample buffer for diluting serum and chromatography buffer. The diagnostic procedure is as follows: dilute the serum samples with sample buffer in the ratio 1:50 and spot an aliquot (5 μL) where indicated by the letter S, and add 100 μL of chromatography buffer at B (Fig. 2a). A red band should always appear at the C line (Fig. 2a) to show that the kit is functional. If positive, a red band appears at the T line within 15 min (Fig. 2a). The intensity of any positive band was estimated visually (unaided) according to the reference card (Fig. 2b). The minimum cutoff level is 0.5. The diagnostic parameters of sensitivity, specificity, and positive and negative predictive values were calculated [30].

## Results

**Fig. 3:**
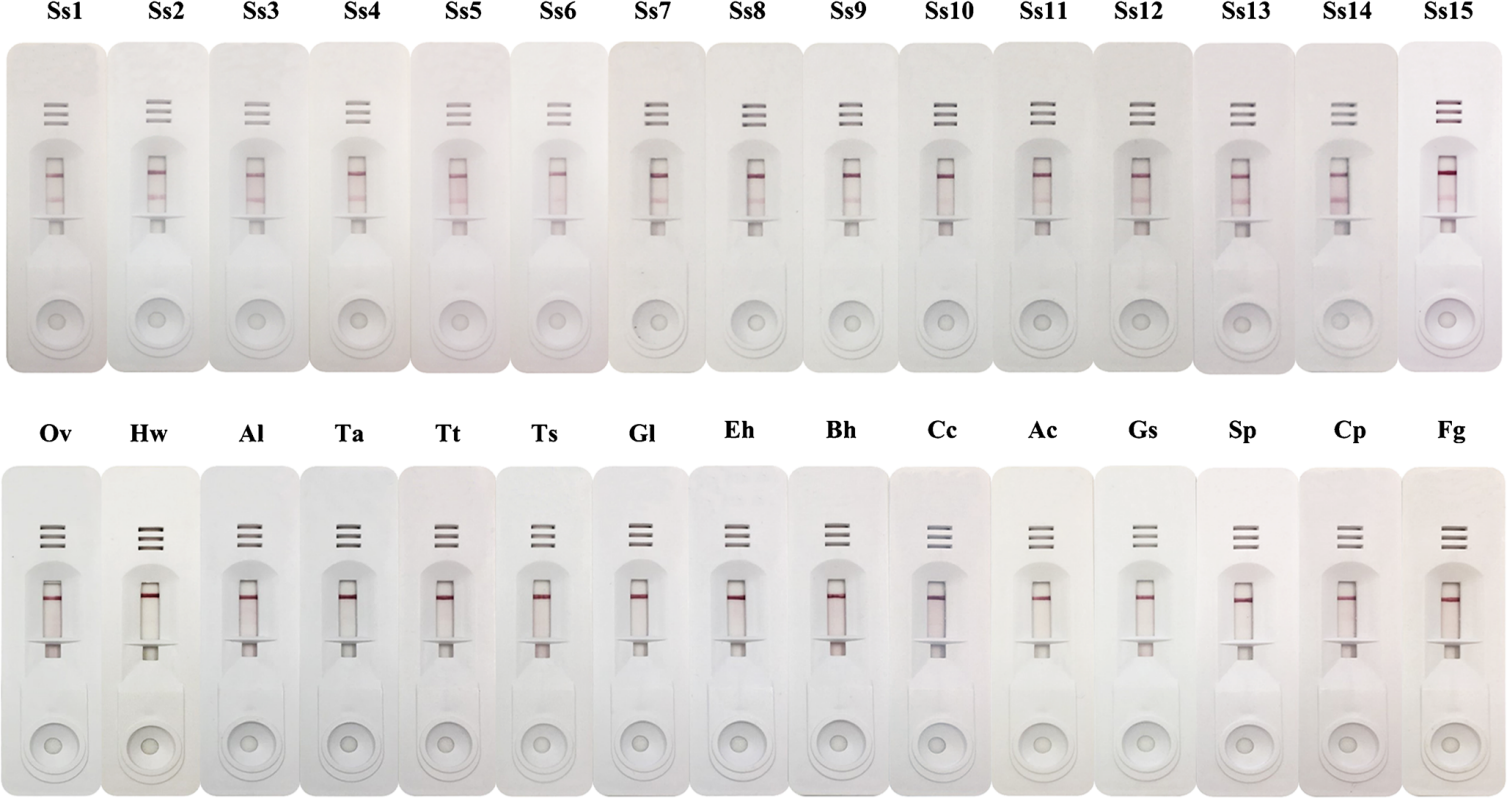
Representative results in ICT kit. Ss1-Ss15, strongyloidiasis; Ov, opisthorchiasis; Hw, hookworm infection; Al, ascariasis; Ta, taeniasis; Tt, trichuriasis; Ts, trichinellosis; Gl, giardiasis; Eh, amoebiasis; Bh, blastocystosis; Cc, cysticercosis; Ac, angiostrongyliasis; Gs, gnathostomiasis; Sp, sparganosis; Cp, capillariasis; Fg, fascioliasis

The strongyloidiasis ICT kit was evaluated using sera from strongyloidiasis patients, healthy controls, and patients with other parasitic diseases (Table 1 and Fig. 3). Fifty-six serum samples (56/60) from the confirmed strongyloidiasis patients yielded positive results using a cutoff value of 0.5. In contrast, none of the 30 healthy control sera showed positive results. Some cross-reactivity was observed in serum samples of hookworm infections (1 of 5), taeniasis (2 of 5), trichuriasis (2 of 5), giardiasis (1 of 5), amoebiasis (1 of 5), blastocystosis (1 of 5), angiostrongyliasis (2 of 5), gnathostomiasis (1 of 5), sparganosis (2 of 4), capillariasis (2 of 5), and fascioliasis (2 of 5). When using 0.5 or above as the cutoff level, the diagnostic sensitivity, specificity, and positive and negative predictive values in ICT were 93.3%, 83.7%, 76.7%, and 95.6%, respectively.

## Discussion

Point-of-care tests offer considerable advantages for the treatment and control of infectious diseases, including parasitosis [31]. To date, there is still no ICT, the POC test, to detect antibodies for diagnosis of human strongyloidiasis. Here, we developed an ICT using *S. stercoralis* larval extract as the antigen for detection of IgG antibodies. The ICT is rapid, simple to perform, and requires no special equipment or skilled personnel for serodiagnosis of human strongyloidiasis.

We did note some cross-reactions with sera from patients with other parasitoses (Table 1). This is not likely to be a real problem in a clinical setting because the clinical presentation of each of these parasitoses differs from those of strongyloidiasis. We should also note that some patients in group iii (those with other parasitoses) may in fact have had asymptomatic strongyloidiasis: these sera were collected in a strongyloidiasis-endemic area in northeast Thailand. The diagnostic efficacy of our test needs to be evaluated further, using samples from countries where *S. stercoralis* is not endemic. Another note should be considered for ICT sensitivity is some strongyloidiasis sera that found *S. stercoralis* by the agar-plate culture method were found negative by ICT (Table 1). This reason be possible due to these serum samples were collected in acute phrase of strongyloidiasis and resulting in low antibody response.

In general, confirmation of strongyloidiasis in chronic carriers is laborious because they usually have a low parasite burden and larvae are released only intermittently in stool [1]. Such asymptomatic carriers can unexpectedly experience dangerous hyperinfection if they become immunocompromised for any reason. Fatal systemic strongyloidiasis can occur in immunosuppressed patients (i.e., patients who receive systemic steroids or cytotoxic treatments (e.g., treatment with anti-neoplastic agents)) [32]. Patients from endemic areas and travelers, who have returned from such areas, should be examined for strongyloidiasis before being given immunosuppressive treatment [33, 34]. Our ICT can be used as the POC test for screening of such patients, and appropriate anti-helminthic drugs are prescribed for positive cases. A further use for our ICT device is to assess cure in clinical care: the ICT antibody reaction should decrease in level through time post-treatment.

In conclusion, we have developed a diagnostic tool that is fast, simple to use, and can supplement stool examination for clinical diagnosis of strongyloidiasis. It can be used at the local level in resource-poor settings (middle- and low- income countries). The method is important for screening asymptomatic-infected individuals and populations who are “at risk” of developing hyperinfection syndrome or disseminated strongyloidiasis if they are given immunosuppressive treatment for other conditions.
